# Understanding Smartwatch Battery Utilization in the Wild

**DOI:** 10.3390/s20133784

**Published:** 2020-07-06

**Authors:** Morteza Homayounfar, Amirhossein Malekijoo, Aku Visuri, Chelsea Dobbins, Ella Peltonen, Eugene Pinsky, Kia Teymourian, Reza Rawassizadeh

**Affiliations:** 1 Department of Biomedical Engineering, Amirkabir University of Technology, Tehran 159163, Iran; m.homayounfar@aut.ac.ir; 2 Electrical and Computer Engineering Department, Semnan University, Semnan 35131, Iran; amirhossein.maleki1990@semnan.ac.ir; 3 Center for Ubiquitous Computing, University of Oulu, 4500 Oulu, Finland; aku.visuri@oulu.fi (A.V.); ella.peltonen@oulu.fi (E.P.); 4 School of Information Technology and Electrical Engineering, The University of Queensland, Brisbane QLD 4072, Australia; c.m.dobbins@uq.edu.au; 5 Department of Computer Science, Metropolitan College, Boston University, Boston, MA 02215, USA; epinsky@bu.edu (E.P.); kiat@bu.edu (K.T.)

**Keywords:** smartwatch, battery, deep learning, convolutional neural network, user experience

## Abstract

Smartwatch battery limitations are one of the biggest hurdles to their acceptability in the consumer market. To our knowledge, despite promising studies analyzing smartwatch battery data, there has been little research that has analyzed the battery usage of a diverse set of smartwatches in a real-world setting. To address this challenge, this paper utilizes a smartwatch dataset collected from 832 real-world users, including different smartwatch brands and geographic locations. First, we employ clustering to identify common patterns of smartwatch battery utilization; second, we introduce a transparent low-parameter convolutional neural network model, which allows us to identify the latent patterns of smartwatch battery utilization. Our model converts the battery consumption rate into a binary classification problem; i.e., low and high consumption. Our model has 85.3% accuracy in predicting high battery discharge events, outperforming other machine learning algorithms that have been used in state-of-the-art research. Besides this, it can be used to extract information from filters of our deep learning model, based on learned filters of the feature extractor, which is impossible for other models. Third, we introduce an indexing method that includes a longitudinal study to quantify smartwatch battery quality changes over time. Our novel findings can assist device manufacturers, vendors and application developers, as well as end-users, to improve smartwatch battery utilization.

## 1. Introduction

Wearable devices are becoming increasingly popular, with smartwatches representing one of the most popular consumer devices on the market. Since 2016, smartwatches have held the third largest market share of wearable devices. This growth rate suggests that the smartwatch market is going to be bigger than other wearable devices and has the potential to grow to the second biggest market by 2022, with an estimated 109.2 million units being shipped worldwide by 2023 [1] (however, it is important to note that this statistic was produced before the Covid-19 outbreak; since the outbreak, the market interest in personal health monitoring devices, such as smartwatches, has significantly increased).

The ability to capture physiological signals (e.g., heart rate) conveniently and continuously and to have quick access to notifications are some of the key features of smartwatches that make them appealing to the public. However, in addition to its advantages [2], every new technology is associated with limitations, and smartwatches are no exception to this. Two well-known smartwatch constraints are the small screen size and limited battery capacity [3]. The small screen makes interaction with smartwatches less attractive compared to other ubiquitous devices, such as smartphones [4]. Furthermore, the size of smartwatches is also directly related to their hardware capabilities, as a small device cannot hold a large battery. Additionally, unlike traditional watches, users have to charge their devices frequently; i.e., every day [5]. As such, the limited battery capability of smartwatches is the biggest challenge restricting its widespread adoption in the market [6]. This issue also affects the usability of these devices; for example, continuously monitoring physiological signals significantly decreases battery power [7]. Moreover, newer versions can work relatively independently of smartphones [3], which requires even more computing power, as the smartphone cannot piggyback on the computational costs of the smartwatch. Therefore, battery optimization is a critical issue for the independent smartwatches that are emerging into the market.

The smartwatch market is expected to grow to $22 billion by 2022. The growth rate of smartwatches has shown the highest increase compared with smartphones, tablets, and laptop ownership, which illustrates the uptake of the smartwatch and penetration of these devices into daily life [8]. A more detailed understanding of smartwatch battery usage could help developers, manufacturers and researchers to design or change their software and hardware for optimal battery utilization. We believe it is therefore necessary to analyze the use of smartwatches in the real world, including different brands and the use of a diverse group of users to gain a holistic overview of their usage. Although promising initial research has been carried out in this field, further research is still required to investigate the significant challenges of this area [5,9,10,11,12]. In particular, research into human–computer interaction (HCI) is a challenge when research goes beyond the laboratory and is carried out in the real world [13]. One issue is that human behavior is multifaceted and complicated, and so more complex models are required to analyze collected data [14,15]. As such, as the complexity of the model increases, our control and accessibility to the model and its functionality decreases [13]. Another challenge is that smartwatches are equipped with low-cost sensors and thus are prone to missing data and artifacts [16], which makes it more difficult to accurately model user behavior. In this paper, we mitigate these challenges with Algorithm A1 for the alignment of sensor data with our deep learning model, which is capable of further exploring the inputs of the model using its first layer filters.

In this work, we benefit from the “insight for wear” (http://insight4wear.com) smartwatch application, which has been designed based on the UbiqLog architecture [17] and has been deployed in the real-world market. This application collects smartwatch data from a number of sensors, including screen usage, heart rate, Bluetooth, light, fitness activity, notifications and battery usage. The dataset, which has Institutional Review Board (IRB) approval, includes different smartwatch brands in different countries with more than 832 users (until July 2019). The analysis is comprised of three segments. The first part utilizes 671 users who have more than 10 days of data for all sensors. The second part focuses on 67 users who have collected data utilizing all sensors for more than 30 days. The third part focuses on 62 users who have more than 6 months of battery data available. Even though not all smartwatch brands include all of the sensors that we have used in our analysis, our method is scalable and appropriate for analyzing all battery-powered wearable devices, including fitness trackers, extended reality (XR) glasses, etc. Within this research, the following questions and related contributions have been posited and discussed:

**RQ1**: “What are the common charging and discharging patterns that users display?” Identifying these patterns can help application developers and device manufacturers to manage smartwatch resources with the aim of less battery utilization. Additionally, these results can help end-users to become more informed about their battery usage and to avoid events that can cause a sudden discharge of the battery. For example, a prediction of the battery consumption rate can be exposed to the end-user at an appropriate time for them to charge their smartwatch before the battery runs out completely [18].

**RQ2**: “What are the discharge peaks, and when do users usually charge their smartwatches (weekday/weekend, time of the day)?” By answering this question, application developers or cloud vendors can optimize their background services or libraries to achieve energy-efficient communication with the network and its applications. By using a pipeline of clustering algorithms, we analyze the battery consumption of devices to identify common patterns of battery utilization based on the time of day. These patterns show the times of the day that users are more likely to charge their devices.

**RQ3**: “What are the drainage symptoms of a smartwatch battery?” We propose a low-parameter fully convolutional neural network (FCNN) model that can be used to identify the correlation among different sensors and their impact on battery usage, using a binary classification of low and high slopes of battery usage. In other words, based on the behavior of the users and sensors, we have designed a deep learning model to investigate if there is any correlation among specific sensors and activities, in terms of battery utilization. By using a simple change in the common architecture of CNNs, our model becomes more interpretable and can be used to extract symptoms from triads, filters and the inputs and outputs of filters. It can also be used in multi-sensor processing (e.g., mobile device sensors) and for different multivariate datasets. The results of our deep learning model also identify the most effective sensors regarding battery consumption and the correlation of sensor data for battery consumption. Furthermore, our model is portable and can be implemented on mobile devices. This is because it is low-parameter and so uses a variety of low-level to high-level features, as well as raw data, as inputs. As such, these features make the model resource-efficient, and so it can be used in on-device data processing.

**RQ4**: “Which brands and operating system versions contribute to a more sustainable battery life?” We benefit from the longitudinal data available in our dataset, which have enabled us to develop an indexing approach that ranks smartwatch battery deterioration based on the brand and operating system version.

The implementation of the deep learning models have been undertaken using the Tensorflow (https://www.tensorflow.org) library and Python version 3.7. To allow for the full reproducibility of our results, all of our code is available in a GitHub repository (https://github.com/mohofar/Smartwatch-battery).

## 2. Related Work

Several research works have focused on smartphone battery utilization [19,20,21,22]. However, battery analysis for smartwatches and other wearable devices is a fairly new area. To provide a detailed overview, we have divided the topic of battery analysis into two categories: smartphones and wearables. In addition, we describe some of the recent developments in deep learning approaches that have been implemented for battery-powered devices.

### 2.1. Smartphone Battery Utilization

Understanding the energy consumption dynamics in smartphones can allow users to better manage their mobile device and can allow developers to better develop their applications; e.g., by caching applications before they launch, etc. However, users are often naive about their battery usage. A study conducted by Rahmati et al. [19] to understand how smartphone users deal with limited battery life illustrated that the average users’ knowledge about configuring their device was insufficient to save power. Falaki et al. [20] have categorized the main factors that cause energy consumption, including the hardware and software platform and the interaction between the application and the user with the device. They have found patterns of interacting with smartphones, and these patterns could lead to correctly predicting the energy loss ratio. Ferreira et al. [21] have found that appropriate feedback based on user behavior can also lead to a reduction in battery consumption. Based on these works, Athukorala et al. [22] have developed an application to inform users of the battery consumption of their smartphones. Their application provides recommendations (e.g., identifying high battery consumption applications) of how to interact with devices to reduce battery consumption. Zhang et al. [23] have presented a power estimation model, which is an automated power model based on the behavior of the battery and uses internal sensors to record battery consumption and is accurate up to 4.1% of measured values for intervals of 10 seconds. This work developed a model that is more generalizable to a variety of smartphones and illustrated that different brands have different power models. However, this work did not directly consider the impact of human behavior on the battery. Mushtaq et al. [24] have also analyzed the effects of software and hardware on the battery consumption of mobile devices in order to reduce it. Their results illustrate that computing in the cloud is an option that can be used to conserve energy.

As well as analysing the device, understanding human behavior is also essential in understanding battery consumption. However, conducting studies in the lab is associated with several limitations that affects the results of user behavior analysis and limits its ecological validity. For example, in the real-world there are many different smartphone configurations that can be used, usage times, applications and usage patterns for similar applications [25], as well as different WiFi signal strengths [9], which are difficult to reproduce in the lab. A hybrid utilization-based and Finite State Machine (FSM) based model that employs real-world data, has been developed by Chen et al. [25] and focuses on energy separation and management. Their dataset was collected using the original Android framework, which did not manipulate the device’s original code (e.g., rooting the phone). As such, this approach may not feasible for real-world deployment. Ding et al. [9] have proposed a model to control the effects of wireless signal strength. Their results show that the energy use for data communication is reduced by 23% for WiFi and 21% for 3G. Furthermore, they identified that location and time of day correlates with changes in signal strength. The energy consumption of different operating system components, such as CPU usage, display and WiFi, was investigated and a model basis for these measurements was presented by Perrucci et al. [26]. Recently, Peltonen et al. [27] have also shown that battery loss where there is limited coverage of WiFi can be 6.29%. These studies show the importance of considering user interaction in relation to their devices’ battery life. However, in contrast to our work, they do not consider the challenges of real-world application deployment.

### 2.2. Smartwatch and Other Wearable Device Battery Utilization

There are promising works that have focused on analyzing the battery of smartwatches [5,6,12,28]. Min et al. [6] considered the behavior of smartwatch users separately, and this separation helped them to quantify the behavioral diversity of smartwatch and smartphone users. As such, through this separation, the results of their study assist in increasing the accuracy of smartwatch user behavior prediction. Jeong et al. [28] analyzed the behavior of wearing smartwatches among 50 students for 203 days and extracted several temporal usage patterns from the data. The results illustrate that there are three factors—contextual, nuanced and multifaceted—which affect wearing behavior. Despite promising results, their research was limited to students and only to one type of smartwatch (Apple watch). Therefore, the generalization of their findings to other smartwatch brands and user groups is contentious.

Based on statistics from 2016, Gartner Inc estimated a 29–30% abandonment rate [29]. A study by Fadhil [30] inspected the motivation behind such abandonment and introduced several reasons, such as lack of usefulness, technical limitations, personal reasons, boredom and device malfunction. Fadhil [30] argued that the motivation behind having a smartwatch can influence the user’s usage and the duration of utilizing it.

Meanwhile, Dehghani et al. [31] analyzed challenges and motivations that lead to purchasing smartwatches. They divided the problem into three important factor—design esthetics, uniqueness and screen size—which explicitly affect the purchase rate of non-users and implicitly affect the frequency of usage and battery consumption of smartwatches.

There are several works [5,10,11] which analyze the physical activity of the user from a wrist-mounted wearable. Liu et al. [5] proposed a model to distinguish usage patterns, energy consumption and network traffic to optimize battery consumption. Their study included 27 participants over 106 days, during which they recorded their smartwatch use. Despite promising results, this study again was limited to one model of smartwatch (LG brand) and a limited variety of users. Furthermore, they averaged power after 10 repetitions of an experience, which reduced the data quality, as real information was lost. These limitations reduced the accuracy of their model for real-world applications. Shoaib et al. [12] analyzed the CPU, memory and battery usage of a smartwatch while the sensors were operating. They then created a model to analyze a target, such as human activity recognition. They reported that sensing and feature engineering consumes most of the smartwatch’s resources, while classification uses fewer resources. In addition, their study reported that using a complex model and using fewer sensors is better than using a simple model with more sensors. Yao et al. [32] explored the predictability of battery consumption for 27 users over 9 months. They reported that the CPU is responsible for 29%, display for 30% and Bluetooth/WiFi for 3.5% of the battery’s drain. Poyraz and Memik [33] reported the importance of the screen and CPU for the use of active power. They provided a power model based on a linear regression model to analyze user behavior, power consumption and network activity. They also reported that third-party applications use the battery more than built-in applications. Visuri et al. [34] used two sensors (notification and screen sensor) from 307 users to quantify smartwatch use in the real world. They reported that smartwatches are used more briefly in contrast with smartphones. However, they analyzed the behavior of users based only on notification and screen sensors. Table 1 summarizes the existing findings that are reported in the state-of-the-art smartwatch research works.

### 2.3. Deep Learning Models for Battery-Powered Devices

Deep neural networks have created significant breakthroughs in computer vision [35], sequential signal processing [36,37], machine translation [38] and many other disciplines. Deep neural networks for mobile sensors are also used in several research projects, including human activity recognition [39,40], healthcare applications [41], user authentication [42], speech recognition [43] and so forth. There are two possible ways to apply deep learning methods to wearable devices: processing the data in the Cloud or edge node or locally on the device. Since our focus is on battery consumption, we omit the first approach [44]. The use of deep neural networks, via the second approach, needs to handle various limitations when used with smartwatches, such as RAM for updating the parameters of the model and speed, due to a lack of parallel processing [45]. It should be noted that our model is not on-device and we only use it to analyze the inputs. Nevertheless, it can be extended for use with on-device applications.

Due to being a black-box data-driven model (e.g., neural networks), there are many works [46,47,48,49,50] that have tried to construct a transparent model to extract semantic information from the model and data. For example, ref. [51] present an up-convolutional network that converts the feature map to an input image. Such an image can help us to identify the parts of the input image that construct the main part of the feature map. However, all of the mentioned models require the addition of new parameters, which increases the computational burden on their hosting device. In this work, we employ a deep learning model to understand smartwatch battery utilization in the real world, without adding any extra parameters and computation on the device.

## 3. Methods

### 3.1. Dataset

We benefit from a real-world dataset that has been collected using the “insight for wear” Android application (http://insight4wear.com/) for smartwatches, which collects users’ data for scientific purposes. The data collection is IRB-approved with end-users’ consent. The dataset consists of eight different sensors, including “screen usage”, “heart rate”, “Bluetooth connection”, “ambient light”, “activity state” (extracted from the Google FIT (https://developers.google.com/fit/) API, including walking, still, in-vehicle and unknown activity), “notification” and “battery usage”. The Google Fit API was utilized to collect the physical activity data of the user, while other sensor data were implemented using native libraries of the Wear OS platform. The details of each sensor implementation and data collection policy are available in work proposed by Rawassizadeh et al. [18], which describes this framework. If the user accepts the sharing of their data with the application provider, the data are uploaded every three hours from their smartwatch to the smartphone. The data are then compressed and uploaded to a central server whenever a WiFi connection is available. In general, data are only stored for 10 days on the smartwatch device; regardless of successful or unsuccessful updates, it will be discarded after 10 days. This policy guarantees the optimization of storage on the smartwatch while reducing the uploading of large packets of data to the network. We have utilized 671 users’ data (out of 832 users) whose battery sensor data are available for more than 10 days. The first stage includes clustering battery utilization using our deep learning model. Users have been selected according to whether their device has collected data from all of the above sensors (67 users) [18]. To rank battery changes during the devices’ life time, based on brand and OS version, 62 users have been selected who have more than six months of battery data available (1,428,743 battery records) for 10 different brands, including Sony, Motorola, LGE, Mobvoi, Huawei, Asus, Samsung, Polar, Tag Heuer and Fossil, and seven different OS versions, including Android 4.4, 5.1.1, 6.0, 6.0.1, 7.1.1, 8.0.0, and 9. Details of the data of selected users are summarized in Table 2. The rest of the users in the datset either did not choose to share their battery data within the app or had less than 10 days of battery data available. In total, we have collected 1,299,782 samples of battery sensor data from 20,306 user per day. Table 3 illustrates a sample of data extracted from the raw data to explain the necessary attributes of the data set. Note that “NaN” values in this table, and other missing data, were converted into a constant number—i.e., zero—for our analysis.

### 3.2. Finding Common Patterns

We employ *k*-means clustering to find common patterns of charging and discharging. Before applying *k*-means, a time-series of the battery charge state from the start of the day (00:00) to the end of the day (23:59) has been extracted. However, due to the irregular sensor sampling intervals of smartwatches, the number of samples (for the extracted time-series data) are not equal. In this instance, the time series data have been re-sampled, using fast Fourier transformation (FFT) [52], to a constant length based on the number of hours per day (i.e., 24) and samples in a day.

We also extract the time of plugging-in and unplugging from the charger (in hours) to analyze the relationship between battery utilization and time of day. Then, we identify the time (hour of the day) of each charger being plugged-in/unplugged for each user. This approach enables us to identify the highest and lowest rate of battery charge or discharge in a day. By using battery utilization gradients (or slopes) instead of the battery state, we can determine when the fastest discharge time of the day occurs. Next, we analyze the average increase in battery consumption on Saturdays and Sundays—i.e., weekends in most countries except in the Middle East and North Africa (which have different weekends)—to see whether there is a difference in battery consumption between the weekend and weekdays. However, we do not consider Friday as part of the weekend for users in the Middle East, which is a limitation of our work. The distribution of the participants is reported in Table 4.

### 3.3. Fully Convolutional Neural Network (FCNN) Model

In order for the battery discharge data to be used within the deep learning model, the data have been transformed into a binary classification problem; i.e., high or low slope.

Each record in our dataset contains the sensor name, sensor data and the timestamp corresponding to the sensor data (as described in Table 2). We convert timestamps (in string format) into numbers; i.e., time IDs. Since the sensor data are not recorded simultaneously, the timestamps are asynchronous. Therefore, our time alignment algorithm (Algorithm A1 in Appendix A) uses the transformed timestamps (time ID) to prepare the inputs for the CNN model.

To train the deep learning model, labels have been generated by calculating the slope of battery consumption in the battery time-series data and dividing these slopes into two classes of high and low battery consumption. This enables us to reduce the battery discharge problem into a binary classification problem. Finally, we normalize the data before analysis by our deep learning model.

#### 3.3.1. Time Alignment

Sensor data were recorded over different times of the day and are therefore not synchronous. Therefore, the number of samples and the time of recording data are not equal for two sensors on the same device. Therefore, it is necessary to align all the timestamps of the recorded data. The time ID consists of a single decimal number, containing the last two digits of the year, month, day, hour and minute, respectively. Furthermore, timestamp alignment allows us to easily sort data objects, based on the transformed timestamps, into time IDs. Therefore, we have added a column of time IDs into the dataset that corresponds to the original record of the data from the sensors. This is similar to the one-hour temporal granularity used in human behavioral analysis to match the timestamps of multivariate sensors [14,53].

We map the type of physical activity (e.g., “still”) to decimal numbers between the “start-time” and “end-time”. Therefore, for the Bluetooth and activity sensors, we utilize the nearest previous point instead of linear interpolation [54]. For the notification sensor, we count the number of notifications between a considered time ID and its previous time ID.

#### 3.3.2. Label Preparation

By dividing the battery status, which is in percentages, into two classes of low and high slopes, we are able to create a classification model to evaluate the impact of inputs on the high and low slopes.

The next step is to assign labels to train our model. To analyze the behavior of battery usage, we calculate slopes between any two continuous points of battery usage data. To calculate slopes, we use the last four digits of the time IDs as hours and minutes. To subtract time IDs, we perform time subtraction, which is limited to 60 (e.g., min) and 24 (e.g., h) instead of mathematical subtraction. Positive slopes have been omitted, as these indicate that the device is charging. As such, the range of negative slopes in our dataset is between −40 and 0. Since the goal of our model is to examine the impact that smartwatch sensors have on the battery discharge rate, an optimal threshold is required to distinguish between the two classes (low and high slopes). It should be noted that the dataset contains many low values, which is indicative of the device being idle. As such, training a network on slopes that contain mostly low values is challenging, as the classes are unbalanced. To mitigate this challenge, we have divided the labels into two classes; i.e., high slopes and low slopes. Our first solution for selecting an optimal slope threshold is to choose the middle of the label based on the average of the maximum and minimum. However, this division causes many data points to remain lower than the threshold, which produces very unbalanced classes. Another solution is to calculate the average of all the slopes. In this case, samples close to the threshold are divided into two classes, although there is no significant difference between them. To select the best boundary point between two classes, we consider the arithmetic means of all data points and the arithmetic means of the maximum and minimum of our data points. We select seven data points between these two points to choose a threshold for the two classes. However, for all the selected points as a threshold, the classes are still unbalanced with a ratio of 0.16 up to 0.000091 for these seven points. Nevertheless, they are more balanced in comparison to the first approach.

**Figure 1:**
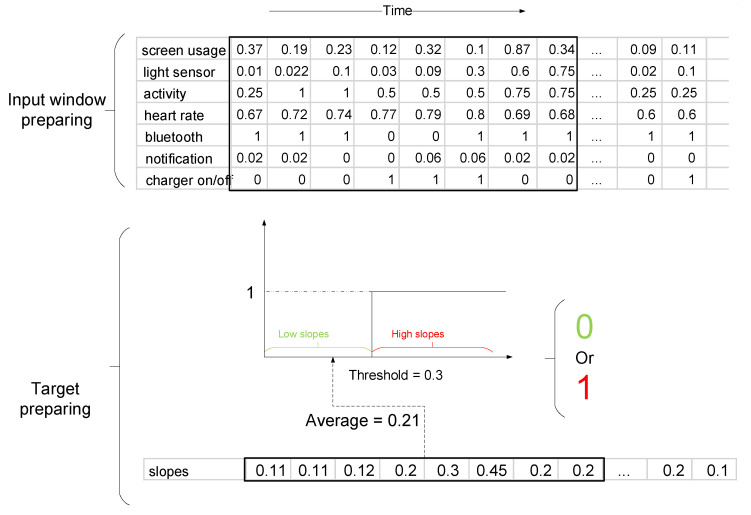
First, at the “ìnput window preparing” stage, seven time-series (sensors data) are concatenated. Next, eight samples (as an example) of each sensor data are selected as the input for our model. The average of eight corresponding slopes are calculated. Next, for quantization, we use a hard-limit transfer function [55], with zero for low slopes (green) and one for high slopes (red) with a border of (0.3).

Moreover, we also need to select an appropriate length of the signal to feed into the deep learning model. The length of the signal has been determined by parameter sensitivity analysis. For example, suppose the appropriate input length is eight samples; for each input matrix with a size of 7 × 8 (or number of sensors × length of sensor data), we have an array with the size of 1 × 8 as corresponding targets (slopes). We need to quantize this array of targets into either a zero or one (as there are two classes). As such, we have employed the average (arithmetic mean) of the target array and have quantized it with a threshold. Figure 1 presents an example of this process.

#### 3.3.3. Input Preparation for the Model

The data from the sensors do not have an equal range and are in different numerical formats. Therefore, z-score normalization [56] has been utilized to prepare the inputs for the FCNN model [57]. The z-score for each signal has been calculated individually based on the “mean” and the “variance” of each signal. However, the signal amplitudes vary, with some being much larger than others. However, since we want to use all the data together in one matrix as inputs, we therefore need to limit the amplitudes of the signals to between zero and one, with a min–max normalization [56]. This normalization takes place after the z-score standardization.

However, the range of the normalized data are not the same as with the first normalization. Therefore, if we want to convolve a filter on all the signals together, the filter weights are presented with a different range of numbers. Therefore, we use the min–max normalization method (as the second normalization) to equalize the range of all the signals. These two normalization processes have been applied to each sensor. For sensors with quantized labels (notification, battery charger on/off, physical activity type and Bluetooth connection/disconnection), only min–max normalization is applied.

#### 3.3.4. Training Phase

In order to train our model, we use the battery status in percentage to label the data (target) and seven sensors’ data (including screen usage, heart rate, Bluetooth state on/off, ambient light, type of activity, number of notification and state of the charging charging/discharging) as inputs (see Table 2), with the length of *n*. Our classification model separates the sensor data into low-consuming (low slope) and high-consuming (high slope) classes. By analyzing the first layer filters of the model, we can identify which sensors have the most important role in low and high-consuming classification. The inputs of this model number seven (number of sensors) × *n* windows. To get the optimum number for *n*, we have examined four lengths: 10, 15, 20 and 25. We do not consider lengths above 25 or less than 5 because smaller numbers contain too little information for classification while larger numbers lead to a significant increase in training time and also consume more memory for training and testing the model. We have identified 5 and 25 thresholds through several iterations of training and testing our model.

The labels of this model are the slopes of the battery utilization, which are 1 × *n* vectors corresponding to windows of the inputs. Since we have to select one slope for every window, we have calculated the average of all *n* numbers of the target array. This means that we can use *n* sensor data to estimate the average of *n* slopes that are low or high (see Figure 1). To use all the slopes, we window all the time-series data with one-sample shifting (red rectangle in the “input window preparing” part of Figure 1).

**Figure 2:**
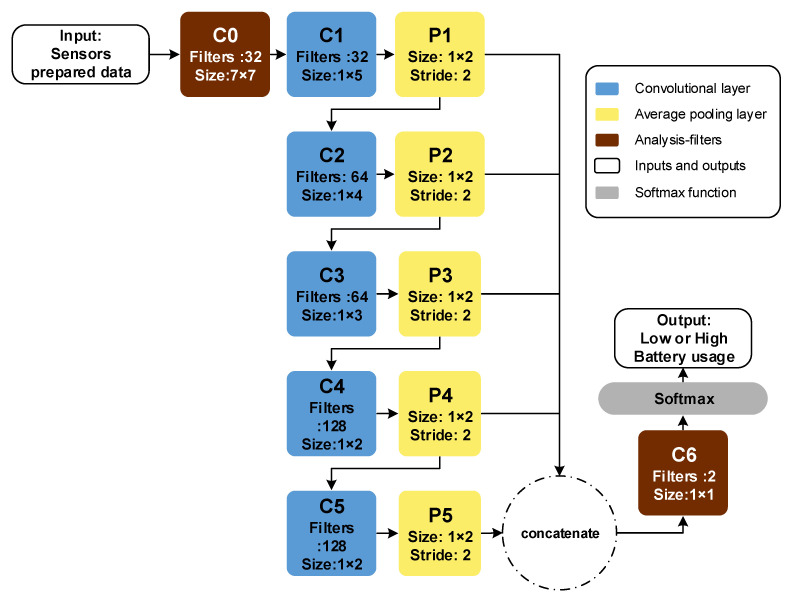
Structure of the fully convolutional neural network (FCNN) model for battery consumption analysis. Convolutional layers are marked with “C” and pooling layers are marked with “P”. Two convolutional layers (C0, C6) are specialized in filtering for analysis. The outputs of the pooling layer are concatenated and fed into the sixth convolution layer to prepare data for softmax function classification.

We then construct a fully convolutional neural network (FCNN) with seven convolutional layers and five pooling layers. Our FCNN includes 1 × 1 filters, instead of a fully connected layer, which reduces the number of trainable parameters [57]. Figure 2 presents the structure of our model.

The first convolutional layer (C0) has three differences from other convolutional layers: first, we eliminate other parameters that depend on the filter convolution of the input and we remove the bias term [35] of the layers; second, 2D filters (7 × 7) of this layer move in one direction on the input (7 × N), meaning that each row is applied to the corresponding input row; third, we do not use zero padding [58] to ensure any row of the filter applies to the corresponding row of the input. Moreover, if we apply seven filters with a size of 1 × 7, we cannot take into account all the interactions of the inputs together.

We call the first and last layer filters analysis filters; as such, the rest of the analysis was performed on the basis of the two described analysis filters. Other parts of the network include 1D convolution and a pooling layer (average pooling is marked in yellow). Furthermore, convolutional layers extract features automatically based on the fed target (zero and one are labels of classes). The first layer extracts low-level features, while the last one extracts high-level features. To select the best level of the features for this task, we concatenate all the outputs of the layers and feed them into the last convolutional layer (C6). By using this method, we can understand which layer has more information for our task. The last layer is a 1 × 1 convolutional layer, which has two main objectives: (i) to scan the concatenated outputs and to find the effect of its filters to classify two classes and (ii) to use a 1 × 1 convolutional layer, instead of a fully connected layer, which has fewer parameters to train. Note that, in our model, the learning rates for all experiments are 0.0005 and all pooling layers are average pooling [59].

To solve the unbalanced class problem, we employ the *k*-means clustering method [60] to balance the classes, instead of using random choice. Since we intend to extract information from the model, we also apply further clustering to the classes with fewer data points to select data points that do not repeat. Note, *k*-means has been used for both of these described clustering approaches. Applying clustering to a class enables the maximum variation to be used, which is useful to train our model.

It has also been recommended to use dynamic time warping (DTW) for time series [61] distance measurements. However, we use Euclidean distance one-to-one mapping as a similarity metric. If we concatenate all sensor data in a window (7 × *n*) to an array, by using the DTW (a one-to-many mapping); the clustering algorithm mistakenly makes a comparison (for assigning an array to a cluster) between a sensor data point and other nearby sensor data in the array. Therefore, we employ the Silhouette coefficient [62] to select the best number of clusters, and for the sake of brevity, we do not report its details here. At this point, our model and data are prepared for training.

#### 3.3.5. Rationale for Introducing a Customized FCNN Model

One can argue about the effectiveness of the development of a customized FCNN model compared to simply using an existing machine learning model. The motivation behind the creation of our model is the superior accuracy that a customized model provides versus existing methods. To demonstrate the superior accuracy of our FCNN model, we compare its results with six well-known classifiers (see Section 4.3). Each of the baseline classifiers uses the same data set for training and testing as our proposed FCNN. These classifiers include Support Vector Machine (SVM), Random Forest (RF), Decision Tree (DT), Naive Bayes (NB), Logistic Regression (LR), and *K*-Nearest Neighbor (KNN). Furthermore, two types of inputs are fed into these baseline algorithms—raw data and extracted features—using our deep learning model.

### 3.4. Indexing Battery Performance

Our dataset includes longitudinal information about battery use. This feature allows us to observe and study the quality of smartwatch battery changes, based on different brands and operating system versions (Wear OS). We have selected users whose data includes more than six months of battery information. As a result, we have utilized 62 users within this analysis. Every day, a time series of battery usage, in percentage, has been created for each user. Therefore, selected users will have at least 60 time series data points of battery use.

**Figure 3:**
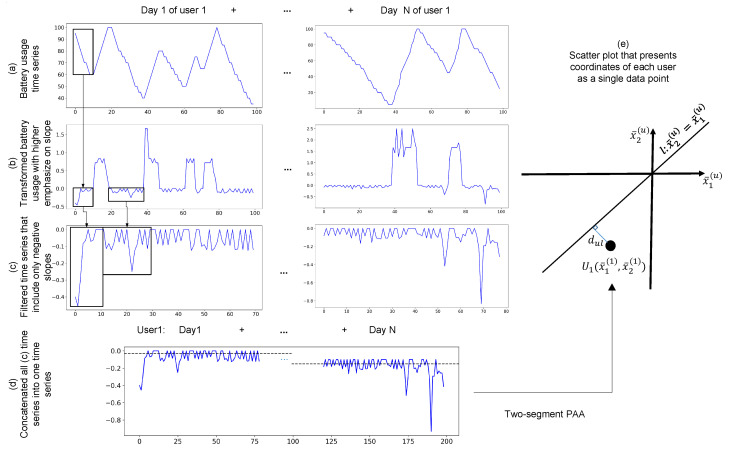
The block diagram for the procedure of indexing battery performance for a user. (**a**) shows the battery status for *n* different days for a user. (**b**)Then, we calculate the slope of plots of from “a” into “b” using their time stamps (**c**) and remove the positive slope in “c” which shows the charging of the device. (**d**) After concatenating negative slopes of “c” for all days of a user, using piecewise aggregate approximation (PAA) in “d”. (**e**) we extract two number of ($x_{1}^{\left(u\right)},x_{2}^{\left(u\right)}$) and make a comparison with the benchmark line ($x_{1}^{(u})=x_{2}^{\left(u\right)}$) in “e”. The mathematical equation for this equation can be found in Appendix B.

To understand users’ battery changes, we have created an index that is a numerical representation of battery changes over time. First, inspired by other works in this area [63], we transform the original time series (Figure 3a) into another time series, whereby the amplitude of changes is the slope of the original time series, see Figure 3b. This is necessary because our goal is to quantify the change in battery discharge, which are slopes of battery status. Therefore, this transformation creates a time series that places a higher emphasis on slopes.

In the second step, we create another time series (see Figure 3c), which removes the charging signals (positive slopes) and only keeps the discharging signals from the time series generated in Figure 3b. In the fourth step, we concatenate all generated signals from Figure 3b, in temporal order, together. The result is shown in Figure 3d, and so each user will end up having one time series of connected slopes. In the fifth step, we use piecewise aggregate approximation (PAA) [64] to transform the new signal into two numeric scalars ($x_{1}^{\left(u\right)}$,$x_{2}^{\left(u\right)}$). In other words, the output of PAA will be the coordinate of the final data point in the scatterplot, as shown in Figure 3e. In particular, one point presets the first ($x_{1}^{\left(u\right)}$) and the other point presents the second half ($x_{2}^{\left(u\right)}$) of battery life for each user (u).

The scatter plot in Figure 3e includes a line, ($x_{1}^{\left(u\right)}=x_{2}^{\left(u\right)}$ benchmark line). If a data point is located on this benchmark line, there is no change in its battery during its lifetime. In other words, the distance of a data point to the benchmark line quantifies the battery changes from its first to its second half life, and the mean of battery discharge does not change from the first half life to the second half life; i.e., $x_{1}^{\left(u\right)}=x_{2}^{\left(u\right)}$. If $x_{1}^{\left(u\right)}>x_{2}^{\left(u\right)}$, then the battery quality is degrading in its second half life; if $x_{1}^{\left(u\right)}<x_{2}^{\left(u\right)}$, then the battery improved in its second half life.

After all battery data have been summarized and transformed into these two-dimensional coordinates, we use the minimum Euclidean distance of the data point from the benchmark line [65]. The data points are then ordered based on this distance. When the second life of the battery improves, the point stays on the top of the list, and when their second life of the battery deteriorates, the point stays at the bottom of the list.

## 4. Results

### 4.1. Patterns of Battery Drain Identified from Clustering

**Figure 4:**
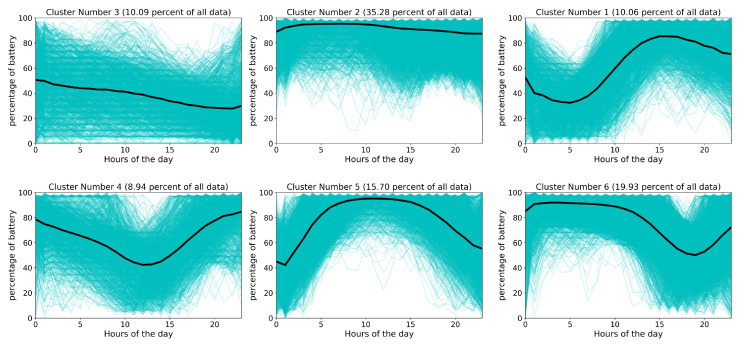
Result of the *k*-means clustering. These plots present the average percentage of battery drain based on the hours of a day. Cyan is used to show time series in the same cluster, and black curves show the center of each cluster.

Figure 4 illustrates the results of *k*-means clustering for battery status, based on the time of day. To find the most appropriate number of clusters, we use the Silhouette coefficient [62], which measures how closely a data point matches the data within its cluster and how closely it matches the data in the neighboring cluster.

Figure 4 shows that 50.98% of users (clusters 2 and 5) charge their device between 1 a.m. and 10 a.m. every day. In total, 19.93% of users charge their smartwatch in the last hours of the day (cluster 6). There are uncommon patterns of charging for about 30% of users (clusters 1, 3 and 4), which show that charging time might happen in different times of the day and that there is no routine pattern.

We also count the frequency of plug-ins based on the day of the week for 671 users. Table 5 shows the summation of all users’ charger plug-ins based on the day of the week. Based on the presented summation in Table 5, there is a difference between Saturday and other days of the week. A one-way ANOVA [66] statistical test (*p*-value = 0.01) indicates the difference is significant.

**Figure 5:**
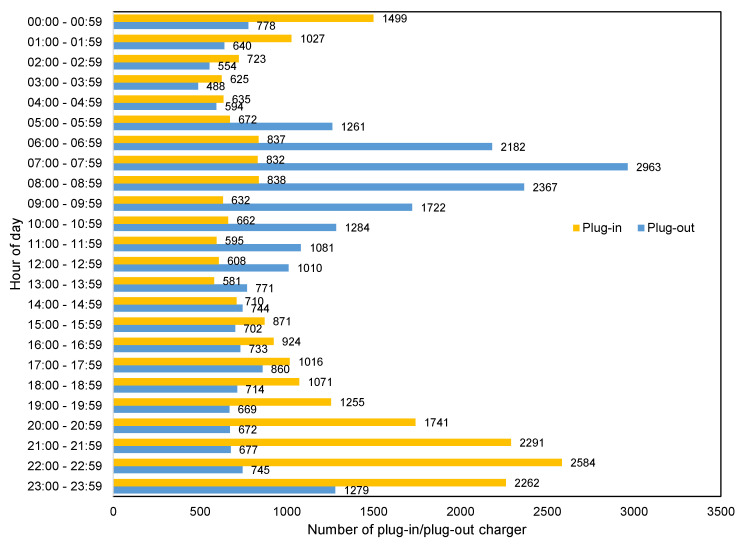
Plug-in and unplugging frequency based on the time of the day.

Figure 5 presents the number of plug-ins and unpluggings based on the time of the day. This figure shows that users unplug and plug-in more often between 6:00–9:00 a.m. and 9:00–11:59 p.m. than other hours of the day; Figure 5 shows the most frequent charging time.

### 4.2. Results of the Deep Learning Model

Our FCNN model focuses on identifying events of high battery consumption. In the described model, we have a specific analysis-filter layer that allows us to find events. First, we find the parameters of the model; then, we explore the model with identified parameters to find out which parts of the inputs were important for the model.

#### Evaluation of the Deep Learning Model

Our FCNN model requires the identification of two parameters: (i) the border of classes and (ii) the length of the input window. Once suitable parameters have been found, we continue with the analysis of the heat maps from the first layer of the FCNN.

To the best of our knowledge, no clear border between low and high battery usage has been previously reported for mobile devices or smartwatches. To identify the best distinction border point, we find two numbers from the average of our data points: 0.069 and the middle of data with the maximum and minimum of data, which is 24.02. We experimented with different border values between 0.069 and 24.02 to select the optimal value for the border of classes.

There is a trade off between the computational cost and accuracy of the model. Short windows suffer from a lack of information, and so accuracy is reduced. On the other hand, a long length of inputs has two major drawbacks: firstly, increasing the length of the inputs and outputs of the layers leads to an increase in the number of calculations; second, if we calculate a label for each window of inputs, averaging can lead to an inappropriate label (loss of labels with high slopes). To prepare the input windows, we use a rectangular window [67], with an amplitude with a size of one. To find an appropriate length for the input of our model, we examine four lengths: 10, 15, 20 and 25 (larger numbers increase the computational cost of the model). Table 6 shows the results of experimenting with these values.

The experimental conditions for all experiments reported in Table 6 are the same. The training and validation data for each experiment have been randomly changed. As shown in Table 6, increasing the length of the input windows results in more information and also increases the training accuracy. The increase in length from 15 to 20 shows a significant improvement in training and validation accuracy. However, increasing the length to 25 is not recommended, as it increases the instability of the validation data and computational cost. Therefore, the model and its parameters are optimal with an input length of 20.

The last layer (layer 6 in Figure 2) of the model contains two 1 × 1 × 640 filters. The output length of the pooling layer, which is concatenated, is 640. The size of the concatenated outputs and filters are equal. Two filters have been applied to the concatenated signal; these filters reduce the size of their inputs into 1 × 1 × 2. We have employed Softmax for the final prediction. All layers of the model have a similar range of amplitudes; therefore, we have kept all the layers to increase accuracy. To evaluate the accuracy of our model, the average confusion matrix of our experiments is reported in Table 7.

The accuracy of the confusion matrix of our deep learning model is 85.33%. In the training phase, 31 input windows were incorrectly predicted as upper borders, and 13 windows were mistakenly identified as being below the border.

### 4.3. Comparison with Other Methods

**Figure 6:**
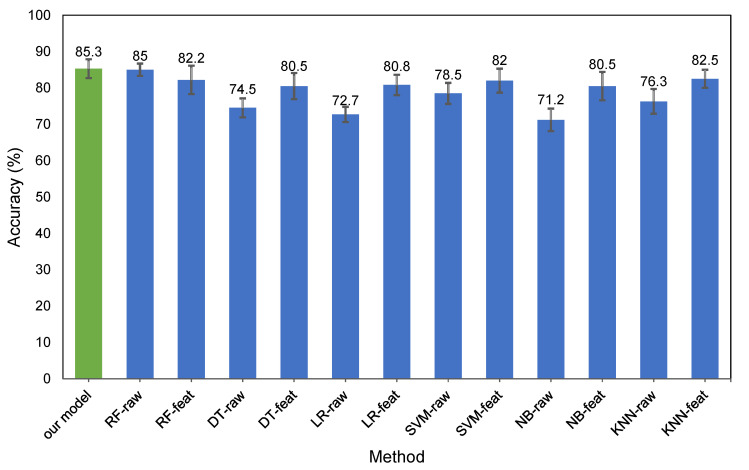
The accuracy of the other methods in comparison to our FCNN model. The abbreviations are as follows—RF: Random Forest, DT: Decision Tree, LR: Logistic Regression, SVM: Support Vector Machine, NB: Naive Bayes, KNN: *K*-Nearest Neighbors. The suffix “raw” refers to inputs in the raw data format and “feat” refers to inputs as features extracted from the FCNN model.

Figure 6 presents the results of the comparison of our deep learning model with other classifiers based on accuracy, showing that our model outperforms other classifiers with raw data. This figure shows that the extracted features from FCNN improve the accuracy of all models, except for Random Forest. In addition to its superior accuracy, another advantage of our model is its ability to find useful features. This is not possible with other machine learning methods, even with Random Forest, which holds a fairly competitive level of accuracy (not using raw data).

### 4.4. Filter Exploration of the Deep Learning Model

**Figure 7:**
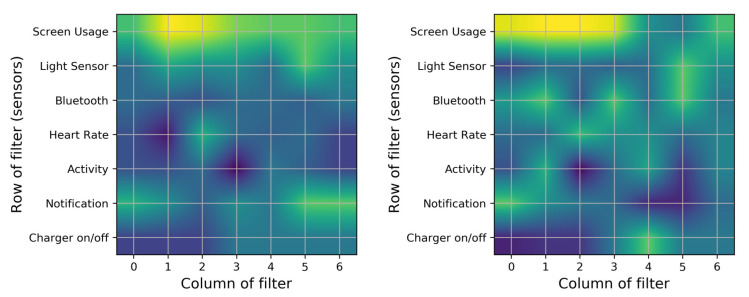
Average of 32 filters’ heat maps for two individual training sessions. The brightest regions highlight the sensor’s focus on extracting symptoms from inputs. The brightest regions show the sensor’s importance.

To investigate our FCNN model further, we have also studied the analysis-filter layer. This layer has 32 filters, with a size of 7 × 7. The input size is 7 × 20 and the output size is 1 × 14 × 32, for each window of inputs. To address RQ3, we have identified the sensors that are responsible for increasing battery utilization. In this instance, we have trained the network and have extracted the trained filters. We have 32 filters, with a size of 7 × 7; if we make a sum of all 32 filters, we will have 7 × 7 filters, which shows the importance of filters. Figure 7 shows the sum of 32 trained filters for two different training samples.

These filters illustrate which part of the inputs were important to consider for the remaining layers of our neural network. The yellow parts present the larger coefficients, which is the aim of our investigation. Based on Figure 7, the screen usage sensor is the most important sensor for these classification tasks, and there is no difference between other sensors. To get a more in-depth evaluation, we perform a sum of the columns in Figure 7, and Table 8 shows the results for five training stages.

In order to understand which inputs are more important for the neural network, we need to determine the most effective filter. Therefore, we have selected one of the training inputs, its parameters and its outputs. The results of this model are reported as a confusion matrix in Table 7. However, to select the most important filter, we select the output of the analysis-filter layer with 300 matrices, with a size of 14 × 32.

**Figure 8:**
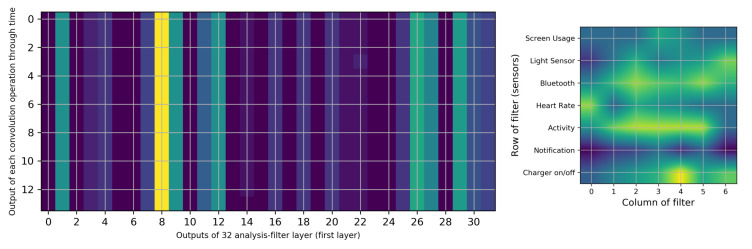
The average of 300 outputs of analysis-filter layers (**left**) and heat map of filter number 8 (**right**).

If we average across all matrices, we obtain a 14 × 32 matrix. This matrix is shown in Figure 8 as a heat map. Figure 8 (left) depicts the 32 columns of the filter outputs and 14 rows of the outputs of the filter convolutions with a stride of one over time. However, filter 8 is different from the others. To answer the next questions (“Is there any correlation among sensors in battery utilization?” and “Is there a certain activity that leads to more battery consumption?” (sub-questions of RQ3)), we extract filter number 8, which is shown in Figure 8 (right).

To understand the behavior of the filters, we have to answer the following question: which changes extracted from the inputs are the most important? Due to the importance of high slopes in this study, we have selected six inputs from the validation to understand the network extracted from the inputs. These inputs are summarized in Table 9.

**Figure 9:**
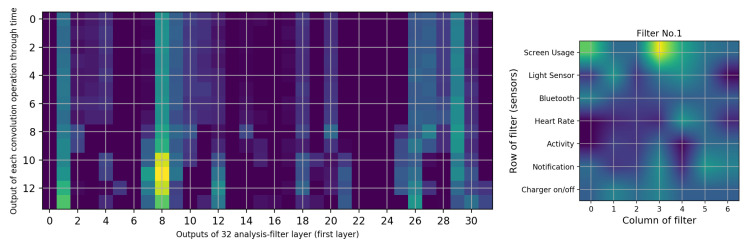
Outputs of analysis-filters for input number 281 (**left**) and heat map of filter number 1 (**right**).

Table 9 lists six different inputs with their targets (1 as high and 0 low slopes) and predicted labels using the neural network and its probability. For each input, we list the important trained filter using the output of the analysis filter (e.g., Figure 8). In the following, we report our analysis with three elements: inputs, filters and outputs. First, for input number 281, outputs of analysis-filters (as shown in Figure 8) are shown in Figure 9.

**Figure A1:**
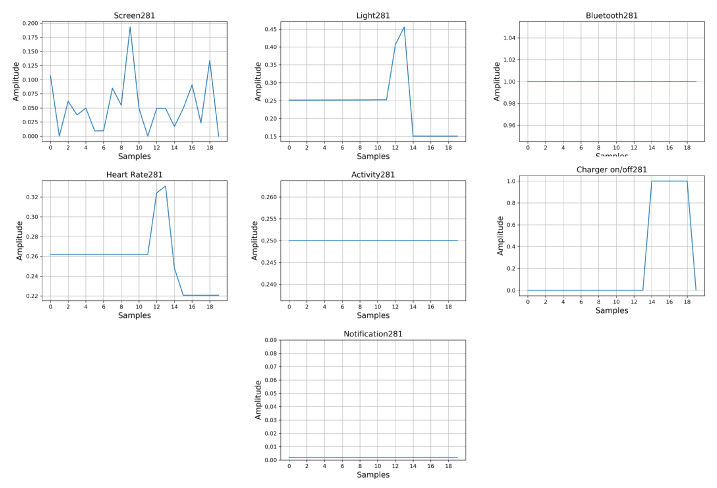
Sample 281 (Screen, light, Bluetooth, heart rate, activity, notification, and charger on/off sensors).

The main role of filter 8 is highlighted in Figure 9 (left) with a lighter color (yellow). Changes in the brightness of columns 8 and 1 can be mapped to the result of the filters. Figure 9 (right) shows filter number 1. For the sake of brevity, the sensors’ time-series for input 281 in Table 9 are shown in Figure A1 in the Appendix C.

Filter 1 (Figure 9 (right)) gives a higher weight to the screen usage sensor than the other sensors. Other parts of this filter have different features; however, since there are no significant differences among them, we cannot make a valid justification for this.

Figure 9 shows that the output of filter 8 has a maximum brightness in the 11th step and around it. We can observe that the Bluetooth sensor is “on” at all times and the number of notifications is constant. Considering the size of the filter and the maximum peaks, two peaks for “heart rate” and “ambient light” sensors are detected. Even after the watch has been connected to a charger, filter 8 reduces the brightness of the output.

### 4.5. Indexing Battery Performance Results

Based on the described method in Section 3.4, we analyze and rank the battery drain over the lifetime of watches for 62 users who have more than six months of data. All the results of the data points on the scatter plot (e.g., Figure 3) are associated with particular brands and Android versions. Smartwatch brands used for our experiment include Sony, Motorola, LGE, Mobvoi, Huawei and Asus. There were other brands in the dataset, including Samsung, Polar, Tag Heuer, and Fossil. However, the users of these brands did not have a minimum of six months of data and so they were excluded from this analysis. Additionally, several other brands were also excluded as there was only a maximum single instance of data. Therefore, we cannot claim that this finding is generalizable to all brands in the market.

The average of $d_{u}$ is an index that is used to make a comparison between different wearables (see Appendix B for more details). In particular, it is used to make a comparison between different smartwatch brands and Android versions, as has been presented in Table 10. By using the $d_{u}$ score, we can make a battery impact comparison based on the brand and operating system version.

Table 10 shows that two brands, Sony and Motorola, perform better in their second life time than other brands, with a small positive $d_{u}$. This means that their battery discharge rate is reduced in the second half of the battery’s lifetime. This could be due to an improvement in their smartwatch skins, or native applications. On the other hand, recent Android versions seem to be less batteryefficient. We believe this is due to the advances in applications that host other applications in the smartwatch, and thus the battery drain is faster. Besides this, we note that the computational capabilities of smartwatch devices did not change, and newer versions of Wear OS were likely to be increasingly more on newer hardware capabilities.

## 5. Discussion

In this work, we have overcome the limitations of previous research [18,28,37,39,41,60] that has mainly focused on a specific user group (e.g., students) [28] or one brand of smartwatch only [28,35]. In addition, none of the previous works considered the impact of all smartwatch sensors on the analysis of the battery. In comparison to previous research, our work utilizes a significantly larger amount of users and a longer duration of recording data. Below, we report the advantages of introducing our novel deep learning model and summarize our main findings.

Identifying common charging and discharging patterns can help application developers and device manufacturers to manage smartwatch resources with the aim of less battery utilization. Additionally, identifying discharge peaks and common charging patterns of smartwatches can aid application developers or Cloud vendors in the optimization of their background services or libraries, obtaining more energy-efficient communication with the network and its applications. Our findings, which are summarized later in this section, are based on the longitudinal data available in our dataset, which have enabled us to develop a method that ranks smartwatch battery deterioration based on the brand and operating system version for application producers.

### 5.1. Advantages of a Deep Learning Model

We have proposed a novel model for analyzing the battery drain in smartwatches. Our model, FCNN, uses all available smartwatch sensors together, which is important because all of the sensors are correlated with battery utilization. As an example of sensor correlation, when a notification arrives, the screen turns on; therefore, we have to consider all sensors together, as investigating sensors individually would change and bias the results. Three reasons to justify our use of the FCNN model are listed as follows:

- First, we need to have an automatic feature extractor based on battery usage, and the most common method is to use CNN. The lower number of parameters of FCNN versus the CNN and multilayer perceptron (MLP) pair lead us to favor FCNN.
- To extract information for the model to learn, we use limited filters of the first and last layers of our model. This functionality cannot be achieved for other models (e.g., RF) that have similar performance in their final decision.
- Residual connections help us to improve the performance of the model by mixing different levels of features to make the final decision.
- By using a binary classification, we can identify whether the extracted information is related to high or low peaks of battery usage. However, other classification methods, such as regression or multiclass classification, cannot identify such a relation. For example, if we use 10-class classification and choose 1 to be the lowest battery usage and 10 to be the highest battery usage, features that are extracted might be related to two low battery usage classes (e.g., 1, 2 and 3). However, we need only high and low battery users (binary selection) and not multiple classes for the selection.

There is promising research (e.g., Min et al. [6]) that mitigates unbalanced data by randomly upsampling the data. However, it has been reported [6] that random upsampling can cause overfitting. We have solved this issue using clustering, which balances the data and reduces the risk of overfitting. Thus, the interpretation of the model is not correct; there is high accuracy but unbalanced data in the classification.

Our model outperforms other classifiers while also providing several advantages that are specific to our model, including the following. (i) In contrast to other deep learning models, our analysis-filter increases the transparency of the models. This is the first step towards solving the trade-off design challenge in HCI [13]. Previous works (reviewed in Section 2.3) have mostly been constructed for image datasets and suffer from excessive parameters and complex computations. (ii) By using a 2D layer for the first layer, we utilize multivariate inputs, which is another advantage of our model. This enables the model to consider the interaction between sensors, and its result is more trustworthy than analyzing each sensor’s impact individually on battery usage. (iii) By using 1D layers in our deep learning model and using the 1 × 1 convolution layer as a classifier, the number of trainable parameters of our deep learning model is very low (4928 trainable parameters). To our knowledge, previous works on smartwatches and smartphones do not have an automatic model to extract information (e.g., the CNN model [57]) to analyze the behavior of users based on the device sensors.

### 5.2. Findings and Recommendations

In the following, we summarize our novel findings that can help device manufacturers, vendors and application developers, as well as end-users, to improve smartwatch battery utilization.

- Devices with newer versions (than 7.1.1) of the Wear OS operating system drain the battery faster than previous versions. Table 10 shows that installing newer versions of Wear OS on the same device causes a deterioration in the battery quality of the device. This could be due to the advances and increase in the number of applications available for smartwatches. All smartwatch devices—without exception—receive updates both from their vendors and Google as an operating system provider. Besides, the number of installed applications does not significantly increase or decrease. Therefore, the number of applications do not play a role in battery discharge.
- Motorola and Sony smartwatch software updates improve battery life, while the battery life of other brands in our dataset deteriorates when updated. Based on the results in Table 10, Sony and Motorola smartwatches have the longest sustainable battery discharge rate, which improved during their lifetime. This illustrates that these two particular brands most likely invest in improvements to their background services and smartwatch skins with the aim of better energy efficiency. Our dataset does not include all existing brands; thus, we cannot claim that this finding is generalizable among all brands.
- The highest peak of smartwatch battery drain is during working hours, from 9:00 to 17:00. On the other hand, sleep time, 22:00 to 6:00, is the best time for batch operations on devices, as most users plug their device in to charge for the longest period of time. Cluster 5 and cluster 2 in Figure 4, which account for more than half of users, show that battery usage increases sharply from morning to evening. The behavior of 15.70% of users (cluster 5 in Figure 4) is similar to cluster 2 but with steeper slopes. Other clusters (1, 3 and 4), which account for 29.09% of all users, have different patterns caused by non-routine days. On the other hand, cluster 6 in Figure 4 shows that approximately 19.93% of users charge their device during the last hours of the day and the first hours of the next day and unplug their device before sleeping. Clusters 3, 4 and 6, which represent about 40% of users, show that most smartwatch users connect their device to a charger at bedtime (i.e., at the end of the day or during the first hours of the day) and remove their device from the charger when they wake-up. Figure 5 shows that most users prefer to plug-in their device during the last hours (before sleeping) and unplug their device in the morning (after waking up). This confirms the result of Figure 3, which illustrates the charging patterns of most users. Based on this pattern, we can recommend that batch jobs, such as backing up data to the Cloud or application updates, which require a great deal of battery power, can be performed during sleeping hours.
- Different charging behaviors are observed on weekends compared to weekdays. Figure 4 shows that there is a difference between Saturday and other days of the week. To evaluate this result, we utilize the results of all users’ data who plugged-in for 7 days of the week. By applying a one-way ANOVA statistical test [66], we found that there is a significant difference between Saturday and other days of the week with a *p*-value < 0.05. A justification for why only Saturday and not Sunday was considered could be due to countries that count Sunday as a working day, such as some countries in the Middle East and North Africa, which biases our results toward Saturday.
- Interaction with the screen and notifications are the most common causes of battery drain. Based on the results presented in Table 8, we identify that screen usage and notification sensors had more of an impact on battery discharge in comparison to other sensors. The heat map presented in Figure 8 shows that this filter extracts features with most focus on physical activity and Bluetooth, then on heart-rate and then on connection to the charger (which turns on the screen automatically).
- When users are physically inactive, there is more battery utilization than during physical activities or when inside a vehicle. The effect of turning the charger on/off can be seen in column 8 of Figure 8. When the charger is connected, the model does not consider any other condition. The screen sensor is on average in its maximum state. The Bluetooth sensor is “on” for all of these periods. The heart rate is constant and at an average which is natural. The light sensor increases to its average of all data of light sensors. In addition to the effect of the charger, the activity type is also correlated; the screen, light, heart rate, and Bluetooth show no related changes. The notification sensor is not important for filter 8 based on Figure 9. Among the four types of activities that the Google service can identify, when the user is inactive, more battery consumption is present than in other states. This finding might appear obvious, but it is important to note that earlier versions of smartwatches have strong false positives with wrist movement and automatically turn on the screen [68]; for example, while the user is driving, moving the steering wheel causes the smartwatch to turn on the screen and thus to drain the battery. Our analyses reveals that this problem has been resolved (More details are available in Appendix C).

## 6. Conclusions and Future Work

In this work, we benefit from a large dataset to identify the patterns and symptoms of smartwatch battery utilization in the real world. We have used a dataset that is composed of 832 smartwatch users, including different geographical regions and brands. In the first stage, we employed the *k*-means clustering method to identify general smartwatch battery discharge patterns. Then, to delve deeper into the battery analysis, we designed a novel low-parameter FCNN, which allowed us to identify the impact that multivariate data (other smartwatch sensors except battery) has on a single source of data (battery). Next, we proposed an indexing approach that enabled us to justify longitudinal battery changes based on the version of the operating system and brand of the device.

We report six novel findings of smartwatch battery utilization patterns, which could help device makers, developers and service providers to improve the battery utilization of their products. In future work, we intend to integrate our findings into a battery manager application on a smartwatch or other battery-powered device, such as a robot, and observe the impacts on the behavior of the battery.

## Figures and Tables

**Table 1:** Summary of findings in state-of-the-art smartwatch battery utilization research.

Paper	Key Findings
Min et al. [6]	- Battery consumption of smartwatches is lower than smartphones - Satisfaction and concerns with smartwatch battery life - Recharging patterns of smartwatches using 17 participants
Liu et al. [5]	- Push notifications, CPU, screen and network traffic are important to battery usage
Shoaib et al. [12]	- Impact of recognizing smoking task on CPU consumption by using one sensor in one task (recognizing smoking) for the smartwatch
Yao et al. [32]	- Predictability of battery usage, application launches and screen display
Poyraz and Memik [33]	- Importance of screen and CPU in use of active power - Third-party applications use the battery up to four times more
Visuri et al. [34]	- Different behaviors between smartphone and smartwatch users - Notifications and screen sensors are used for their analysis
Jeong et al. [28]	- Analyzing temporal wearing patterns of smartwatches - Studying wearing behaviors of smartwatches

**Table 2:** Dataset description.

Sensor Name	Total Number of Data Points (671 Users)	Numbers of Data Points (67 Users) for the Deep Learning Model	Description
Screen usage	2,340,760	489,756	- Start and end time, when the screen is on or off
Heart rate	380,099	101,287	- Beats per minute (bpm) based on user-defined intervals for recording
Bluetooth	506,545	42,822	- The timestamp for establishing or disconnecting a connection from the smartphone via Bluetooth
Ambient light	1,104,511	355,660	- Ambient illuminance (lux)
Activity	502,176	80,948	- Type of activity (extracted from the Google FIT API)
			- Activity duration
Notification	10,965,195	594,157	- Name and time stamp of the notification package
Battery	2,599,564	266,667	- Battery status in percent
			- State of charge (charging or discharging)
Total number	18,398,850	1,931,297	

**Table 3:** Example illustration of sample data extracted from the raw data. For “Fitness Activity", “1” stands for an unknown activity, “2” is for Walking, “3” is for Still, and “4” is for In-Vehicle. “min” stayed for minutes and “lux” for illuminance.

Time Stamp (Original Format)	Fitness Activity	Number of Notifications (Normalized)	Battery Sensor	Bluetooth	Screen Usage (min)	Heart Rate	Light Sensor (lux)
10608291121	1	0.001	56.5	1	0.13618333	71	750.167
10609201429	1	0.001	47.4026	0	0.0823	64	86.7037
10611040931	3	NaN	72.3096	1	0.29395	97	107.556
11611241736	1	0.001	87.0794	1	0.07845	85	NaN
11704291950	1	0.01	89.9823	1	0.07145	86	NaN
14602041950	3	0.1	32.55	1	0.08293333	62	5.41002
18803220806	1	0.02	92.3661	1	0.01396666	83	2
26610211632	1	10	75.6452	1	0.04478333	74	153.889
40702051322	4	0.01	68.0398	1	0.06195	66	28.5085
52706201954	1	NaN	59.6354	1	0.3446	59	2

**Table 4:** This table illustrates the diversity of users across the world by nationality and region. These users’ data contain information about their connections and longitude.

Region	Users (in Percentage)
Europe	47.17
North America	40.75
Asia	10.65
Australia and Ocean	0.94
South America	0.16
North Africa	0.01

**Table 5:** Number of the charger plug-ins according to the day of the week.

Day of Week	Number of Plugged-In Chargers
Sunday	2746
Monday	2824
Tuesday	2798
Wednesday	2952
Thursday	3054
Friday	2840
Saturday	5529

**Table 6:** Details of experiments into choosing the length of input windows.

Input Length	Amount of Data Lower than the Border of Classes (Slope = 0.3) after Clustering	Accuracy for Five-Time Repeat (Mean ± Variance)		Experiment Condition
		Train	Validation	
10	8434	73.2 ± 0.16	70.0 ± 1.2	Batch size = 300
15	5380	77.2 ± 2.96	72.0 ± 1.2	Number of epochs = 200
20	3793	94.24 ± 0.83	**85.30 ± 2.1**	Learning rate = 0.0005
25	3156	96.36 ± 0.46	83.59 ± 3.88	Validation data number = 300

**Table 7:** Average confusion matrix, where five repetitions have been utilized with randomly selected data.

	Lower Border	Upper Border
Predicted lower border	TP = 129	FP = 13
Predicted upper border	FN = 31	TN = 127
TR = true positive, FP = false positive		
FN = false negative, TN = true negative		

**Table 8:** Summation of columns of heat maps.

Sensors	First Training	Second Training	Third Training	Fourth Training	Fifth Training	Mean
Screen usage	16.75	17.07	18.89	20.02	18.76	**18.30**
Light sensor	13.50	13.95	13.86	12.69	12.61	13.32
Bluetooth	13.24	14.53	12.72	12.81	11.79	13.02
Heart rate	13.78	13.82	13.43	11.96	12.67	13.13
Activity	13.90	13.58	12.56	12.11	13.00	13.03
Notification	14.30	13.61	15.52	16.92	16.83	15.44
Charger on or off	14.49	13.41	12.98	13.45	14.32	13.73

**Table 9:** Examples of prediction for seven selected validation inputs.

Input Number	Target	Predicted	Probability of Prediction	Slope	Important Filter Numbers
281	1	1	99%	0.62	8, 1
240	1	1	99%	0.66	8
190	1	1	72%	0.30	8
129	0	0	97%	0.1	8
92	1	0	95%	0.71	8, 1
46	0	1	74%	0.06	8

**Table 10:** Average of $d_{u}$ score based on the brands and Android version.

Brands	Average $d_{u}$	Wear OS Version	Average $d_{u}$
Motorola	0.004	5.1.1	−0.004
LGE	−0.009	6.0.1	**−0.0002**
Asus	−0.026	7.1.1	−0.0209
Huawei	−0.033	-	-
Sony	**0.009**	-	-
Mobvoi	−0.01	-	-

**Figure A2:**
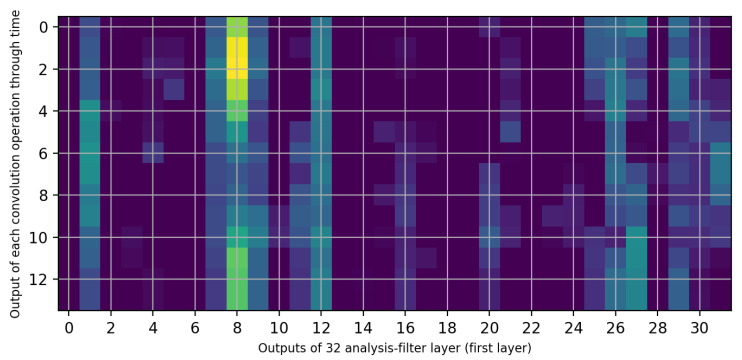
Outputs of analysis-filters for an input number of 240).

**Figure A3:**
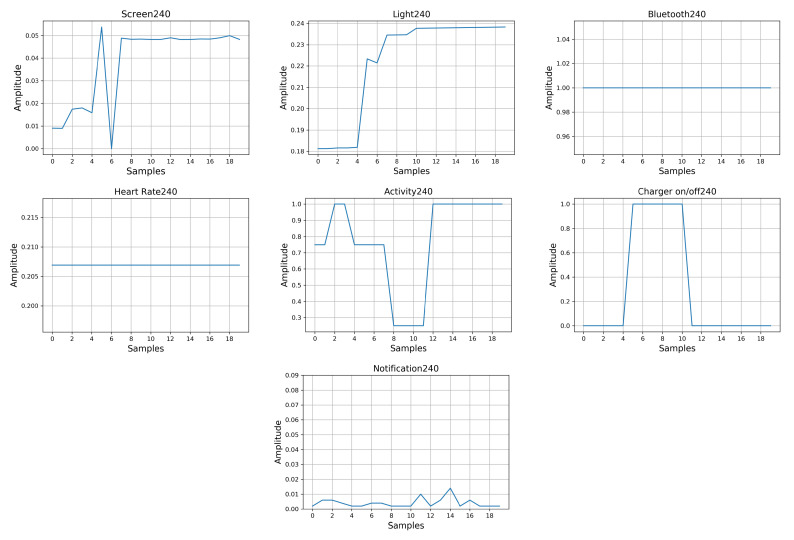
Inputs of input user 240 (screen, light, Bluetooth, heart rate, activity, notification and charger on/off sensors).

## Appendix A

Algorithm A1 shows the process of our time-alignment approach.

**Algorithm A1:** Time alignment of all sensors with one reference sensor

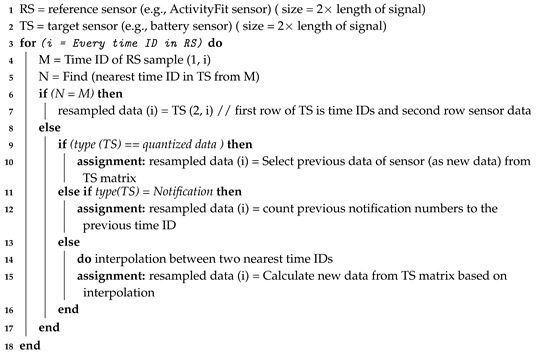

In this algorithm, a sensor is selected as a reference sensor (RS). In our implementation, the RS sensor is the screen sensor, and other sensors are target sensors (TS); see lines 1–2. Then, the algorithm searches for the nearest time IDs for interpolation (line 5), except quantized data, including notification (lines 11, 12), charger on/off, type of activity, and Bluetooth connection (lines 9, 10). For these sensors, the algorithm selects the previous nearest data points (line 10), instead of the interpolation. For the notification sensor, it counts the number of notifications from the previous time ID (line 12).

## Appendix B

Assuming *N* is the number of battery discharge events, each signal result will be converted into a two-dimensional coordinate (Figure 3d). For given battery discharge events *x* = ($x_{1},x_{2},\ldots ,x_{N}$), we can calculate the i th segment for user *u* by using the PAA from *N* samples of *x* events, as presented in Equation (A1).

(A1) $${\bar{x}}_{i}^{\left(u\right)}=\frac{2}{N}\sum_{j=(\frac{N}{2})\left(i-1\right)+1}^{(\frac{N}{2})i}x_{j}\;;i=1,2$$

We can extract two segments, $U({\bar{x}}_{1}^{\left(u\right)},{\bar{x}}_{2}^{\left(u\right)}$), for each user (u), which reduces the time-series data of the battery discharging slope into two numbers (or a point in scatter plot of Figure 3e). When ${\bar{x}}_{1}^{\left(u\right)}={\bar{x}}_{2}^{\left(u\right)}$, this indicates the battery discharging rate is constant.

To perform indexing, we calculate the distance of $d_{ul}$ using Equation (A2) [65].

(A2) $$line-pointdistance:d_{ul}=\frac{|{\bar{x}}_{1}^{\left(u\right)}-{\bar{x}}_{2}^{\left(u\right)}|}{\sqrt{2}}$$

To calculate the overall distance, we use Equation (A2), which presents a minimum Euclidean distance of the data point from the line and is always positive. In addition to the distance, the sign (above or below the line) is important, and so Equation (A2) has been changed as follows:

(A3) $$d_{u}=\frac{\left({\bar{x}}_{1}^{\left(u\right)}-{\bar{x}}_{2}^{\left(u\right)}\right)}{\sqrt{2}}$$

Note the subscript 1 and 2 for $\bar{x}$ are for the first and second segments based on Equation (A1) (for two segments), and thus it needs to be considered that the second segmentation of data has to happen after the first segment. Using Equation (A3), we can calculate $d_{u}$ for each user (u). Now, using this equation, we can order the brands of smartwatches or Wear OS (formerly known as Android Wear) versions by indexing using this equation.

## Appendix C

As another example, for an input number of 240, the outputs of the filters and their inputs are shown in Figure A2 and Figure A3. Figure A1 shows that the screen usage data spikes upward and downward around the average of screen usage of all data. As shown in Figure A3, the last seconds of screen input have a higher value than others. Higher values in Figure A1 correlate with the brightness that we observe at the last points of column 1 in Figure A1.
